# Social Media, Public Health Research, and Vulnerability: Considerations to Advance Ethical Guidelines and Strengthen Future Research

**DOI:** 10.2196/49881

**Published:** 2023-12-29

**Authors:** Philip M Massey, Regan M Murray, Shawn C Chiang, Alex M Russell, Michael A Yudell

**Affiliations:** 1 Department of Community Health Sciences Fielding School of Public Health UCLA Los Angeles, CA United States; 2 College of Education and Health Professions University of Arkansas Fayetteville, AR United States; 3 Department of Health Behavior Texas A&M University College Station, TX United States; 4 Recovery Research Institute Massachusetts General Hospital and Harvard Medical School Boston, MA United States; 5 College of Health Solutions Arizona State University Phoenix, AZ United States

**Keywords:** research ethics, social media, vulnerable populations, public health, ethical guidelines, algorithms, manipulation

## Abstract

The purpose of this article is to build upon prior work in social media research and ethics by highlighting an important and as yet underdeveloped research consideration: how should we consider vulnerability when conducting public health research in the social media environment? The use of social media in public health, both platforms and their data, has advanced the field dramatically over the past 2 decades. Applied public health research in the social media space has led to more robust surveillance tools and analytic strategies, more targeted recruitment activities, and more tailored health education. Ethical guidelines when using social media for public health research must also expand alongside these increasing capabilities and uses. Privacy, consent, and confidentiality have been hallmarks for ethical frameworks both in public health and social media research. To date, public health ethics scholarship has focused largely on practical guidelines and considerations for writing and reviewing social media research protocols. Such ethical guidelines have included collecting public data, reporting anonymized or aggregate results, and obtaining informed consent virtually. Our pursuit of the question related to vulnerability and public health research in the social media environment extends this foundational work in ethical guidelines and seeks to advance research in this field and to provide a solid ethical footing on which future research can thrive.

## Introduction

In October 2021, the Senate Subcommittee on Consumer Protection, Product Safety, and Data Security convened a hearing titled “Protecting kids online: testimony from a Facebook whistleblower” [1]. While this hearing focused on the protection of children, highlighting the amplification of content related to eating disorders targeting teenagers as well as the platform’s “blind eye” toward age verification, broader takeaways included the platform’s ability to create and cultivate a manipulative environment on social media. In part, this senate hearing, which examined questions about the inner workings of the social media ecosystem, was spurred by the 2016 election scandals [2], the spread of misinformation during the COVID-19 pandemic [3], and the mental health crisis that has most notably affected our young people [4]. While public health efforts have played a critical role in combatting misinformation on social media as well as addressing the mental health crisis, little has been done to examine the fundamental question that prompted congress’ interest in social media: can social media create a manipulative environment that makes us vulnerable to undue influence? The short answer is yes, as documented by congressional hearings [1,5-7], independent research [8-11], and investigative journalism [12-15].

## Algorithms and Vulnerability

The driver behind the discussion of social media manipulation appears to hinge on one key idea: algorithms. While research has examined how algorithms create an inescapable environment and thus an extensive network primed for digital discrimination, systematic bias, unethical targeting, and misinformation and disinformation campaigns [16-18], we still do not know enough about how algorithms function, what goals inform these functions, or the impact of algorithms on public health research and practice.

While algorithms and their ethical concerns have entered various dialogues, from congressional hearings to investigative journalism, little discussion has taken place in the field of public health. To date, public health ethics has focused largely on practical guidelines for writing and reviewing social media research protocols. Such ethical guidelines have included collecting public data, reporting anonymized or aggregate results, and obtaining informed consent virtually, to name a few. Susser [19] extends these considerations by discussing the role of manipulation, autonomy, and bias for digitally targeted public health interventions. Ethical considerations when using social media for public health research must expand alongside our increased understanding of how the social media environment functions, specifically concerning the presence of algorithms and how these may contribute to issues related to vulnerability. Privacy, consent, and confidentiality are important hallmarks for ethical frameworks in social media research [20], but we must move beyond these foundational questions and begin unpacking how our research and practice may or may not contribute to and benefit from the manipulative environments that many experience on social media.

The use of social media in public health has advanced the field dramatically over the last 2 decades. Traditional public health methods in surveillance and outbreak investigation [21], approaches in health education and promotion [22], and strategies in policy advocacy [23] and community organizing [24] have all been applied, refined, and adapted for the social media environment. Social media research, or the process of using social media data to conduct quantitative or qualitative research, ranging from observational data collection to experimental designs, is an invaluable tool in public health research and practice and continues to expand both in terms of how it is conducted and where it takes place [20]. Decades of applied public health research in the social media space have led to more robust surveillance tools and analytic strategies, more targeted recruitment activities, and more tailored health education [21]. However, more must be done to advance our understanding of algorithms and how they may ultimately compromise data for public health research and practice.

The potential use of compromised information in public health research is relevant to both observational and intervention research on social media. For instance, when conducting observational research that collects public data from individual accounts related to a specific topic (eg, vaccine safety), how do we disentangle the extent to which content was shared due to behind-the-scenes platform manipulation (ie, due to algorithms that place content in a user’s thread with the goal of increasing interaction and engagement and with little regard to the content itself)? Adding to this complexity is the presence of social media bots, or automated programs, that artificially amplify or spread content based on an array of goals (eg, to spread disinformation, notify of emergencies, share advertisements, or aggregate news articles) [25].

Similarly, for intervention research, do we know how interacting with social media content produced for a research study may influence the platform’s tailoring of future content for that same individual (eg, joining a vaccine-related research study may place the individual at greater risk of being exposed to future highly engaging vaccine information, which is more often than not misinformation)? Intervention research often takes place in closed or “private” groups on social media, making it easier to moderate and monitor the content directly administered by the study; however, the closed group exists within the larger ecosystem of the platform, and we do not yet know how participation in a research study may impact content exposure outside of that closed environment. Furthermore, when using social media to recruit study participants, we must also consider the potential collection and use of compromised information. For example, how much do targeted recruitment ads rely on interactions by users with content that was manipulated or artificially placed in a user’s thread to solicit interaction? These questions highlight very practical ways in which our seemingly innocuous research activities (eg, public data collection or targeted recruitment) may in fact be interacting with and relying on compromised information, thus creating a scenario where researchers are relying on information that is, to an undetermined extent, artificially manipulated by opaque algorithmic intervention, contributing to vulnerabilities that have yet to be considered by public health research taking place on social media.

## Moving Forward

We wish to move the field of public health and social media research forward by posing the following question: how should we consider vulnerability when conducting public health research in the social media environment? We pose this question not to limit or stifle public health research in the social media environment; in fact, quite the contrary—we pose this question to activate our collective understanding and consciousness to strengthen research in this environment in part due to the ever-changing social media landscape. At its core, the primary goal of the social media ecosystem is to keep users on the platform, interacting and engaging with content, for as long as possible, often at any cost [16]. Our hope is that the issues raised here will do the following: (1) contribute to frameworks that more clearly describe how vulnerability, much like privacy, consent, and confidentiality, is an essential concept for conducting ethical social media research; and (2) establish the need for partnerships with social media companies, supported through federal resources, that will facilitate collaborative yet independent research led by academic partners. While algorithms themselves are not nefarious, it is the intent behind the use of algorithms, and the goals and parameters set forth to use the algorithms in particular ways, that evokes concerns surrounding manipulation that may contribute to and enhance various vulnerabilities.
